# To disclose or not to disclose? Mental health service users’ and practitioners’ views of practitioners’ own self-disclosure of mental health difficulties: A mixed-methods study

**DOI:** 10.1371/journal.pmen.0000062

**Published:** 2025-04-08

**Authors:** Kimberly Carter, Nicola Moran

**Affiliations:** School for Business and Society, University of York, York, United Kingdom; PLOS: Public Library of Science, UNITED KINGDOM OF GREAT BRITAIN AND NORTHERN IRELAND

## Abstract

Mental health practitioners’ self-disclosure of their mental health difficulties to service users is increasingly relevant as mental health services move away from dominant biomedical approaches towards relationship-centred care. Yet, this area is under-researched. This paper reports on research undertaken using an explanatory sequential design with 83 mental health practitioners and 68 mental health service users taking part in an online national survey in England, with five practitioners and five service users (none known dyads) then taking part in semi-structured telephone interviews to discuss their views and experiences in greater depth. The study found that mental health practitioners’ self-disclosure could provide a valuable contribution to service users’ care. Self-disclosure offered benefits for both practitioners and service users, such as promoting recovery, facilitating interactions and balancing power differentials; however, stigma remained an issue within the mental health workforce. There was a notable discrepancy in the (perceived) rationale for disclosure between practitioners and service users, and in the way psychiatrists in particular perceived and were perceived to perceive self-disclosure. The findings suggest that practitioners are more likely to disclose the longer they have been practising, suggesting that team culture, confidence and professional capability are influential. There is a need for reflective supervision and clear guidance around self-disclosure, alongside an ongoing drive to challenge stigma, so that practitioners with lived experience of mental health problems are empowered and supported around their disclosure for the benefit of service users.

## Introduction

Mental health conditions are common with one in four people having experienced difficulties at some point in their lives [1]. This suggests that lived experience is also widespread throughout the mental health workforce. However, despite a high prevalence of mental health conditions and trauma history experienced by mental health practitioners [2], many are reluctant to disclose and seek help for their mental health in comparison to physical health conditions [3]. For example, a study by Boyd et al (2016) found that only 16% of mental health practitioners disclosed their mental health problems to their colleagues, despite perceiving lived experience as an asset [4]. This suggests that practitioners lack confidence in disclosing due to a fear of the negative consequences, such as stigma and potential job loss [5].

Studies have considered ways in which disclosure can affect a service user’s relationship with their practitioner. It is indicated that a positive therapeutic rapport predicts better short- and longer-term outcomes [6] and while this could support practitioner disclosure there are concerns it may negatively impact on the practitioner-client relationship. Freud outlined the main reasons for therapists not to disclose to their clients, noting fear that disclosure may become directive or shift the focus from patient to therapist [7]. Other psychoanalysts perceived practitioners’ self-disclosure as having a detrimental impact on the therapeutic alliance and saw value in distancing personal interventions from psychoanalysis, however some therapists suggest that expressing personal emotions enables a practitioner to participate more fully in analysis and come to know their own fears and transferences in greater depth [8]. In the midst of this uncertainty, discussion and guidance surrounding self-disclosure is lacking.

Mental health practitioners’ self-disclosure of mental health issues to service users is increasingly relevant as mental health services move away from medical approaches, and towards recovery-orientated care [9]. The benefits of self-disclosure are widely reported, such as building the foundations of a strong therapeutic relationship, promoting empowerment and removing power differentials [10,11]. It has been demonstrated that practitioners valued their lived experience as a resource through which they could assist others and promote service delivery [12]. Lived experience has been found to be foundational to supporting recovery and generating partnership and empathy which can increase patient satisfaction and clinical outcomes [13,14].

Research also suggests that self-disclosure can be advantageous for practitioners. One study proposed that sharing lived experience was beneficial when the experiences were shared by individuals in respected roles [15]. Another study identified differences between professions, with psychologists, occupational therapists and counsellors more accepting of self-disclosure, while nurses, doctors, and social workers viewed self-disclosure as inappropriate and unhelpful [14]. A study of GPs suggested that disclosure by doctors to patients could have a negative impact on primary care doctor-patient interactions [14,16]. These conflicting findings may be influenced by how self-disclosure is treated by management and within professional codes of conduct.

The benefits of practitioner self-disclosure for service users are also captured in research. Some studies suggest that service users view disclosures that are relevant and responsive to their circumstances as positive [17]. Other findings indicate that service users mainly supported the concept of professionals sharing lived experience and perceived mental health as the most helpful topic of disclosure, however it was shared the least often [14]. Whilst this research contains useful findings with a rare consideration of the experiences of service users, the reasons against self-disclosure and a consideration of mental health disclosures were unexplored.

A further study focused on the role of lived experience in mental health education and practice as perceived by social work and nursing students who had experienced mental distress [10]. The findings suggested that personal experiences can inform practice by breaking down the unattainable perception of invincibility among practitioners. However, the study captured only the perspectives of a small population and excluded the experiences of service users.

Research also demonstrates the potential pitfalls of self-disclosure, such as issues around over-sharing, blurring roles and confusing boundaries [17]. Findings suggest that therapist disclosure can generate boundary issues, can diminish perceived competence, and can compromise the client’s view of therapist and client roles [17]. Practitioner disclosure may alter boundaries unfavourably, leading to reduced credibility and confidence in their professional abilities [18]. In addition, staff are concerned that self-disclosure may impact upon career progression, as well as being fearful of experiencing stigma from management [19]. This presents a potential dilemma for practitioners in which if they disclose to service users, they may be required to disclose to management, causing ethical dilemmas in terms of supervision, as well as a fear of loss of registration from regulatory bodies [14]. Ethicists warn that self-disclosure is not compatible with the professional role and therefore warrants a risk management approach [20].

There is limited guidance around the process of self-disclosure within professional work and training [12,14]. While all professions have a document that addresses the need to maintain boundaries, most do not mention self-disclosure [14]. Those that do focus on personal details with little mention of mental health experiences. There is no suggestion within existing professional guidance that self-disclosure constitutes a boundary crossing [14] and it is not prohibited.

Thus, despite a plethora of studies which have looked at different aspects of self-disclosure across social and professional settings and a consideration of the benefits and risks, the subject remains controversial with little consensus. There is a lack of training and guidance to support practitioners when making decisions around self-disclosure and the experiences of service users are largely unknown. As lived experience is becoming increasingly valued within mental health settings, the current research intended to offer greater insight into both service users’ and mental health practitioners’ views and experiences around self-disclosure which may help to inform policy and practice.

This paper reports on a national study conducted in England exploring whether practitioners’ self-disclosure can provide a valuable contribution to the care a service user receives. The research question was thus ‘what are the views and experiences of service users and practitioners around mental health practitioners’ disclosure of their own mental health conditions/diagnoses to service users?’

Specifically, the study explored service users’ and mental health practitioners’ (perceived) reasons for practitioners’ disclosing their own mental health experiences, the response of colleagues and supervisors/managers to disclosure, perceptions as to whether disclosure benefitted service users in the short term and longer term, and the impact on practitioners of their self-disclosure. Three objectives were to (1) gain a picture of service users’ and practitioners’ views and experiences of disclosure through a national survey, (2) explore those views and experiences in depth through qualitative follow-up interviews, and (3) use the qualitative data to help interpret and analyse the quantitative data. It was hoped that this would increase awareness of disclosures that are happening and how these are experienced, and potentially also lead to recommendations around disclosure of mental health problems by mental health practitioners.

## Materials and methods

### Research design

The study was designed to capture the experiences of two populations: mental health practitioners who have disclosed their mental health difficulties to a service user, and service users who have experienced a mental health practitioner self-disclosing. A mixed methods explanatory sequential design [21] was used to first collect quantitative data on the experiences and views of a larger number of mental health practitioners and service users via national online surveys and then to collect qualitative data from a sample of practitioner and service user participants via interviews in an attempt to explain or interpret the quantitative data, develop a deeper understanding of experiences of disclosure and allow for a triangulation of findings to check validity [22]. This design enabled the research objectives to be addressed.

Online cross-sectional surveys, one per population group, were selected because they offer a means of collecting data from a larger number of participants, thus potentially capturing a broad scope of experiences in a cost-effective way [23]. They are also relatively quick to complete and can be undertaken at a time and place of the respondent’s choosing, which is preferable for sensitive subjects [24]. Qualitative interviews enabled the researchers to explore the views and experiences of practitioners and service users in greater depth to gain a deeper, richer, understanding of the nuanced issues around disclosure. Interviews were semi-structured to ensure that whilst all interviewees were asked core questions, there remained scope to probe responses and for participants to raise additional issues that were important to them [22]. Due to Covid-19 lockdown measures in place at the time of data collection (April-August 2020), interviews were conducted by telephone. Although telephone interviews tend to be shorter than face-to-face interviews there is evidence that they produce rich descriptive data [25] and can make participants feel more comfortable in sharing accounts of sensitive experiences [26]. However, telephone interviews can create difficulties with establishing rapport and omit the observance of non-verbal cues such as facial expressions and body language which can change how something is perceived [27].

### Eligibility and recruitment

To be eligible to take part in the survey, participants had to be based in the UK, over the age of eighteen and be either a mental health practitioner (current or previous) who had self-disclosed to a service user, or a service user (current or previous) who had experienced a disclosure from a mental health practitioner. All participants had an opportunity to volunteer to take part in a follow-up telephone interview to discuss their experience in more depth.

Recruitment to the study was national, though the sample was not nationally representative. Participants were recruited online via adverts posted on social media (Facebook, Twitter, Reddit, LinkedIn), blogs, online forums for mental health support groups and professional groups, and bulk emails sent to national voluntary, peer-support and mental health services with requests for them to be distributed through their membership, as well as through the networks of the authors. All had national reach. Recruitment materials were not available in languages other than English and forums and groups aimed specifically at minority ethnic groups or culturally diverse groups were not targeted due to lack of resources. These are limitations of the study. However, it might be assumed that mental health professionals from diverse backgrounds practicing in England might access/read/follow some of the major online groups and forums published in English. More generally, whilst online recruitment can reach individuals that are geographically dispersed and increase response, disadvantages can include a lack of opportunity to clarify the meaning of questions and access issues [28].

Clicking on the survey link in the recruitment blurb took potential respondents to an information sheet explaining what participation would involve, outlining the eligibility criteria and the risks/benefits of taking part. Participants were required to check a box confirming their agreement to a series of written consent statements in order for the survey questions to appear. The surveys, open during Spring 2020, took 5-15 minutes to complete.

At the end of the survey, participants could check a box to express interest in taking part in a follow-up interview. Those who did were emailed an information sheet and consent form about the interview and were contacted to invite any questions and arrange an interview. Participants completed written consent forms and returned them via post or email to the researcher.

Twenty-four practitioners and ten service users volunteered to take part in interviews. In this small-scale exploratory study, ten participants was considered sufficient to produce rich data for analysis [29]. A purposive sampling frame was thus used to select five participants from each group based on their experiences as reported in the surveys. The sampling frame included: a mix of positive, negative and mixed experiences; a selection of professional backgrounds; a variety of mental health services and geographical locations; a selection of different diagnoses/conditions; and differential amounts of time spent working within or accessing services. Interviews lasting 30-60 minutes were held in Summer 2020 and audio-recorded with consent.

### Materials

#### Surveys.

The questions and statements in the cross-sectional surveys were developed by the researchers, based on the research question and objectives and informed by the academic literature. The survey for practitioners was piloted with two practitioners from a local mental health trust; the survey for service users was informed by and piloted with members of the University’s service user advisory group. Surveys and interviews were piloted with the same individuals. Pilot data was not included in the final sample.

Each survey contained 25-30 structured questions. Questions were predominantly closed and forced response with the exception of diagnosis in which multiple answers could be selected. Response options for closed questions were of the form ‘Yes, a lot’, ‘Yes, a little’, ‘No, not at all’ and ‘Unsure’; or consisted of a 7-point Likert scale with options ranging from ‘Strongly agree’ to ‘Strongly disagree’. The surveys sought to obtain a picture of service users’ and practitioners’ views and experiences around disclosure thus questions asked about: the number of disclosures, diagnosis/symptoms disclosed, (perceived) rationale for disclosure, (perceived) impact on the practitioner and any (perceived) short and long-term benefits of disclosure for the service user. Participants were also presented with a series of statements, for example about the impact of the disclosure on the therapeutic relationship, recovery journey, and professional boundaries, and were asked to select the response on a Likert scale which most closely matched how they felt.

The surveys included questions about basic demographics only (gender, age, and, for practitioners, profession and team). This was a conscious decision in the research design as, while we understand that the race or ethnic background and identity of the practitioner and service user could certainly impact on the decision to self-disclose to service users, self-disclosure to supervisors and the wider team, as well as potentially how that disclosure is received, the complexities are vast. Questions about the ethnic background/identity of the practitioner and service user would have meant participants making assumptions/presumptions of their practitioner’s/client’s ethnic background. Even if the practitioner and service user were of the same ethnic background they may have different cultural beliefs, language, religious beliefs, experiences, etc. We would also need to consider intersectionality. The decision to disclose or feelings about receiving the disclosure could be impacted by ethnicity and/or gender and/or sexual orientation and/or disability (to the extent evident) or things that could not be captured such as being fans of the same football team or singer, rapport built over a shared interest/liking of a particular book or TV programme or views on something in the news/world of celebrity etc. We would have had to categorise very different people together for the sake of analysis and that would have seemed false and unhelpful. Nevertheless, we recognise that racism, heterosexism, cissexism, transphobia etc can shape self-disclosures and how people make sense of them.

In addition, there were six optional open-ended questions in the practitioner survey and four in the service user survey. The open-ended questions effectively offered opportunities for respondents to explain their answers in more detail so they could provide more meaning to their responses that in turn would support interpretation and analysis of the survey findings. Research suggests that open-ended questions enable participants to freely construct their responses, provide further detail or explanation and may give a more accurate reflection of participants’ thoughts or perspectives [30]. During the pilot phase, respondents were keen to add thoughts in relation to those particular closed questions and did not feel that any further open questions would be necessary or helpful for the survey stage of the research.

In the practitioner survey, optional open-ended survey questions were: ‘What was the reason for your disclosure?’; ‘Please explain your response (optional)’ following the closed question ‘Would you be concerned that your disclosure could be perceived negatively by your colleagues and manager?’; ‘If you discussed your disclosure within supervision please describe the response you received from your supervisor or organisation’; ‘Please explain your answer (optional)’ following the closed question ‘Do you think your disclosure benefitted the service user in the short term?’; ‘Please explain your answer (optional)’ following the closed question ‘Do you think your disclosure benefitted the service user in the longer term?’; ‘Is there anything you would like to add?’.

In the service user survey, optional open-ended survey questions were: ‘What do you think was the reason for their disclosure?’; ‘Please explain your answer (optional)’ following the closed question ‘Do you think their disclosure was a benefit to you in the short term?’; ‘Please explain your answer (optional)’ following the closed question ‘Do you think their disclosure was a benefit to you in the longer term?’; ‘Is there anything you would like to add?’.

The surveys concluded with an opportunity to enter a prize draw and an option to volunteer for a follow-up telephone interview to explore their responses in more depth.

#### Interviews.

Separate, albeit similar, topic guides were produced for service users and for practitioners. The topic guides were developed alongside the survey questions and adapted once the survey data had been analysed so that interviews could focus on key issues emerging from that data. The topic guides aimed to explore decision-making around disclosure, the experience of disclosure, and any impacts of disclosure on both practitioners and service users. Questions were tailored to interviewee’s responses in the survey to explore any particular issues or insights raised in the survey and/or to explore further what disclosure might mean in relation to a particular team or profession. Questions covered perceived advantages and disadvantages of disclosure, emotional impact, professional boundaries, power differentials, organisational context and whether participants felt the experience may have been different if the professional background or diagnosis of the practitioner or service user was different.

### Ethical implications

All potential participants were provided with an information sheet and opportunities to ask questions about the study prior to taking part. The survey would only open if participants checked a box to confirm that they agreed with the consent statements. Interviewees provided written informed consent via the completion of consent forms, returned either by post or email. The research involved participants discussing difficult times in their lives which could cause emotional distress [31]. Details for support services were thus provided within the information sheets. The survey could be terminated at any time and interviewees were told they could take a break or terminate the interview at any point without giving a reason.

The interview transcripts, open text survey responses and direct quotations were anonymised to remove any potentially identifying information, and demographic data was reported in aggregate to maintain anonymity. Participants who entered the prize draw or volunteered for interview were asked to provide an email address. Prize draw winners were selected at random. Prior to analysis, contact details were removed from the survey and securely destroyed. The study was granted ethical approval by the sponsoring University (SPSW/MTA/2019/14).

### Data analysis

The quantitative survey data was analysed and reported using descriptive statistics. Responses were compared between practitioners from different professions and between those who had accessed/worked for longer within services. Data from open-ended questions in the survey were analysed using content analysis which enables verbatim responses to open questions to be coded into a relatively small set of meaningful categories which can then be analysed [32]. This analysis highlighted the most common types of response to open-ended questions and also indicated the range of responses provided. Qualitative data from responses to open-ended survey questions were analysed using content analysis [33]. Free text data was decontextualised to identify meanings and create a code list, then recontextualised to include content, categorised into homogeneous groups then compiled to enable realistic conclusions to be drawn. All stages were conducted by KC and triangulated and checked by NM. It was not possible to probe or to discuss survey responses with participants hence there was potential for misunderstanding by the participants and researchers, however, open survey questions were few and piloting found them to be clear.

Interview transcripts were analysed by the first author using the framework approach to thematic analysis [34]. The initial coding frame reflected the interview questions and was adapted as emergent codes were identified. Transcripts were coded and recoded as the coding frame was refined. A sample of practitioner and service user transcripts were also coded by the second author. There was a high degree of overlap in the coding and identification of themes between the authors with minor discrepancies being discussed, the coding frame refined and the transcripts re-coded. The coded data were summarised and charted in Excel for clarity of writing up [35].

The quantitative and qualitative findings were then analysed together to explore whether the qualitative data could help to interpret and explain the quantitative data. The findings are thus presented together, quantitative data first, followed by qualitative data, in an attempt to explain what was happening within the dataset, using an explanatory sequential design [36].

### Reflexivity

The lead researcher (KC) was a student social worker in a mental health team when the research was undertaken as part of her dissertation for her Master’s degree. This was clearly stated in the study information sheet. The co-researcher (NM) was the supervising academic with decades of research experience in qualitative, quantitative and mixed methods research. In some respects KC was an insider as she was a student in one of the professions that the study was recruiting from. However, this was a national online study and did not recruit directly from the team or organisation within which KC was on placement. There was potential for ambiguity in power differentials in the interviews. Although KC was the interviewer, the practitioner research participants were more experienced and thus held more professional power, yet participants may have felt more vulnerable, or indeed stronger, as they had already disclosed in the survey that they had experience of disclosing mental health problems. Similarly, service user participants had disclosed accessing mental health services and their diagnosis in the survey. KC was conscious of being open, transparent, non-judgemental, professional and human throughout the interviews and reflected with NM if anything had caused her any concern or impacted on her own emotional state or perceptions of practitioner roles. NM and KC were aware of the potential for unconscious bias but consciously checked themselves and one another through regular discussions to minimise the potential for bias to influence the interpreting or reporting of data [37].

## Results

### Sample

#### Quantitative data: Survey participants.

Eight-three practitioners from across England took part in the survey, of which 70 (84.3%) were female, a majority were aged 26–40 years (n = 48, 57.8%), the most frequently reported professions were community psychiatric nurses (n = 21, 25.3%) and social workers (n = 20, 24.1%), and Community Mental Health Teams were the most frequently reported team (n = 34, 41.0%). Sixty-eight service users from across England also took part in the survey, of which the majority (n = 54, 79.4%) were female, and the most frequently reported age category was 26–40 (n = 36, 52.9%), with a range of 18–65 years. Community Mental Health Teams were the most frequently accessed service (n = 30, 44.1%). Service users’ survey responses showed that community psychiatric nurses and support workers had been most likely to disclose their own MH problems (n = 13, 19.1% each) while social workers disclosed the least (n = 4, 5.9%) (see Table 1).

**Table 1:** Demographics of the survey sample of practitioners and service users; profession and team of those who disclosed their own MH difficulties; and frequency of MH disclosures by MH practitioners.

	Practitioners (n = 83): N (%)	Service users (n = 68): N (%)
**Gender**		
Female	70 (84.3)	54 (79.4)
Male	13 (15.7)	11 (16.2)
Other/prefer not to say	0	3 (4.4)
**Age**		
18–25	9 (10.8)	16 (23.5)
26–40	48 (57.8)	36 (52.9)
41–55	21 (25.3)	13 (19.1)
56–65	5 (6.0)	3 (4.4)
**Profession of respondents (practitioners) or of practitioners who disclosed their MH to service users (service users)**		
Community psychiatric nurse	21 (25.3)	13 (19.1)
Social worker	20 (24.1)	4 (5.9)
Psychiatrist	7 (8.4)	5 (7.4)
Inpatient nurse	5 (6.0)	5 (7.4)
Support worker	5 (6.0)	13 (19.1)
Psychologist	5 (6.0)	11 (16.2)
Occupational therapist	2 (2.4)	5 (7.4)
Other	18 (21.7)	11 (16.2)
Not sure	0	1 (1.5)
**Team worked in (practitioners) or accessed (service users)**		
Community mental health team	34 (41.0)	30 (44.1)
Early intervention service	9 (10.8)	0
Hospital inpatient service	9 (10.8)	6 (8.8)
Child and adolescent mental health service	3 (3.6)	3 (4.4)
Forensic service	3 (3.6)	0
Psychological therapies service	2 (2.4)	11 (16.2)
Substance misuse team	2 (2.4)	0
Assertive outreach service	1 (1.2)	0
Crisis resolution and home treatment team	1 (1.2)	3 (4.4)
Eating disorders service	0	3 (4.4)
Other	19 (22.9)	12 (17.6)
**No. of service users disclosed to (practitioners) or no. of mental health practitioners who disclosed their mental health problems (service users)**		
1	9 (10.8)	35 (51.5)
2–3	36 (43.4)	23 (33.8)
4–5	16 (19.3)	6 (8.8)
6–10	7 (8.4)	4 (5.9)
11–20	4 (4.8)	0
21+	11 (13.3)	0

#### Frequency of mental health disclosures by mental health practitioners.

Survey data showed that most practitioners in the sample had disclosed their mental health difficulties to more than one service user (n = 74, 89.2%), with the most frequently reported number being 2–3 service users (n = 36, 43.4%). The majority of service user respondents reported that only one mental health practitioner had disclosed to them (n = 35, 51.5%), with seven being the highest number of disclosures a single service user had experienced (see Table 1).

#### Qualitative data: Interviewees.

Five practitioners and five service users who took part in the survey also took part in in-depth interviews. Practitioners comprised nurses, occupational therapists, social workers, and psychologists. The demographics of the practitioner interviewees broadly reflected those of the survey participants: four were female and aged 18–55 years, three had been in practice for 0–5 years and two for 11–16+ years, and workplaces included early intervention, inpatient, forensic and primary care services. Service user interviewees ranged in age from 26–65 years, and three were male. The most frequently accessed services were Community Mental Health Teams and Inpatient Services.

### Themes

Analysis of survey and interview data identified six themes: rationale and considerations around disclosure; experienced advantages and disadvantages of disclosure; emotional impact; diagnosis and stigma; professional roles, boundaries and the balance of power; and organisational context. Within each theme, quantitative survey data is presented first (where applicable) and then is explained or interpreted by reference to qualitative survey data (where provided) and qualitative data from the interviews.

#### Theme 1: Rationale and considerations around disclosure.

Practitioners were asked to share their rationale for disclosure via an open-ended survey question. The most frequently reported rationales were to validate, to empathise with and to provide comfort to service users. These reasons were explored further in the practitioner interviews with, for example, one noting that disclosure was deeply embedded within their practice:

*“You know I’m aware you’re not to be best friends [laughs] with the person you work with but it’s a balance between boundaries and disclosure. I personally cannot envisage my job without both of those two things.” (Practitioner 4, Interview)*

The interview narratives indicated that greater professional experience and confidence supported self-disclosure:

*“I think that’s down to maturity and doing the job for a lot of years and maybe knowing how to verbalise things better than when I was twenty.” (Practitioner 5, Interview)*

Context and timing were perceived as fundamental factors when practitioners considered disclosing. A number of practitioner narratives were saturated with nervousness and over-justification when explaining their disclosure:

*“I felt I had a justification as to why it was done if questioned on it…” (Practitioner 3, Interview)*

However, others felt that, in a helping profession, disclosure would be a positive if it were deemed helpful to service users:

*“As a nurse by background or anybody to be fair, we come into this profession to help others and I think you know if by me disclosing some information can help others move forward by giving them a little reassurance then erm… that’s positive.” (Practitioner 5, Interview).*

The mental health presentation of a service user was also deemed an important consideration. Consideration of risk and crisis-planning were deemed imperative amongst professionals considering disclosure. The paramountcy of ensuring the focus was on the client and not attaching too much emotion to the disclosure, particularly in times of distress, were highlighted:

*“When someone’s in crisis you need to talk about them.” (Practitioner 1, Interview)*

#### Theme 2: Advantages and disadvantages of disclosing.

**Advantages of disclosing:** In the survey, the vast majority of practitioners and service users agreed with the statements that it is helpful for service users when a practitioner shares their mental health difficulties (Table 2).

**Table 2:** Practitioner and service user views on the benefits of practitioners disclosing their mental health difficulties to service users.

	Strongly agree n (%)	Agree n (%)	Somewhat agree n (%)	Neither agree nor disagree n (%)	Somewhat disagree n (%)	Disagree n (%)	Strongly disagree n (%)
**Practitioner views (n = 83)**							
I think the service user opened up to me more as a result of telling them about my own MH difficulties	27 (32.5)	30 (36.1)	18 (21.7)	6 (7.2)	0	2 (2.4)	0
I think the service user felt inspired by my own personal recovery from MH difficulties	18 (21.7)	28 (33.7)	20 (24.1)	15 (18.1)	0	2 (2.4)	0
I think a MH practitioner’s self-disclosure to a service user can provide a valuable contribution to the care that the service user receives	26 (31.3)	25 (30.1)	24 (28.9)	7 (8.4)	1 (1.2)	0	0
I think my own experiences of MH difficulties have increased my understanding of MH difficulties in service users	45 (54.2)	25 (30.1)	11 (13.3)	0	2 (2.4)	0	0
**Service user views (n = 68)**							
I opened up to the practitioner more as a result of them telling me about their own MH difficulties	18 (26.5)	8 (11.8)	10 (14.7)	12 (17.6)	4 (5.9)	7 (10.3)	9 (13.2)
The disclosure improved my relationship with the practitioner	14 (20.6)	13 (19.1)	14 (20.6)	10 (14.7)	6 (8.8)	4 (5.9)	7 (10.3)
I thought more highly of my practitioner after their disclosure	18 (26.5)	10 (14.7)	10 (14.7)	16 (23.5)	5 (7.4)	3 (4.4)	6 (8.8)
I think the practitioners’ own experiences of MH difficulties will have increased their understanding of MH difficulties in service users	34 (50.0)	14 (20.6)	7 (10.3)	5 (7.4)	4 (5.9)	2 (2.9)	2 (2.9)
I think a MH practitioner’s self-disclosure to a service user can provide a valuable contribution to the care that the service user receives	26 (38.2)	15 (22.1)	10 (14.7)	8 (11.8)	3 (4.4)	4 (5.9)	2 (2.9)

Most practitioners felt their disclosure benefitted the service user, though they were more confident of this in the short term (n = 77, 92.8%) than longer-term (n = 60, 72.3%) (S1 Table). Similar numbers of service users perceived the disclosure as having a benefit in the short-term (n = 44, 64.7%) and long-term (n = 39, 57.3%).

Interview data provided more detail with some practitioners noting how first-hand experience was felt to offer a deeper level of compassion and heartfelt interactions than textbook knowledge:

*“… you’re talking from your own experience and not something you’ve read from a textbook” (Practitioner 1, Interview)*

Lived experience of mental health difficulties could therefore be considered a valuable part of a practitioner’s identity.

Service users similarly felt that self-disclosure provided openness and vulnerability, which was preferable to a recognisable professional narrative used with client groups:

*“You begin to recognise a certain script in people… especially therapists.” (Service User 5, Interview)*

The extent to which service users reported disclosure as advantageous or helpful to them in part depended upon where they were in their own recovery journey. For some service users disclosure instilled hope and acceptance:

*“She spelled out what life for a person that was in remission looked like… I had no idea that people with mental illness/mental conditions could have a successful life, while still battling symptoms.” (Service User, survey response)*

However, other service users stated that at the time of the disclosure they were not in the right place in their recovery to hear this information:

*“… especially in the early days I would have wanted absolutely none of them to be honest because I was all in me own head and I didn’t want to deal with anyone else’s problems…” (Service User 2, Interview)*

**Disadvantages of disclosing:** Concerns about a comparison between the recovery journeys of practitioners and service users was a consistent theme within the survey and interviews. Despite practitioners believing that service users did not feel judged in relation to their own recovery, some uncertainty was reflected within service user responses.

In the survey, 68 practitioners (82%) reported that they did not judge the service user’s recovery journey compared to their own and also they did not believe service users were worried that the practitioner felt they should be doing better in their own recovery journey (S2 Table). However only 26 (38.2%) service users reported feeling that the practitioner did not judge them in this way with the same number reporting feeling that the practitioner thought they should be doing better.

Interview data suggested that the risk of a shift of focus from the needs of the client to those of the professional was another consistent concern, as well as a risk of transference of emotion if the professional had not sought closure from their difficulties:

*“…all sorts of thoughts went through my head… I did hold it together erm and I was fine, but I guess from a professional point of view, those are the things you have to weigh up because we are human at the end of the day and you’ve got to be mindful that you’re not adding to their plate…” (Practitioner 5, Interview)*

Other narratives referred to a potential role reversal in which the service user may feel obligated to support the practitioner thus placing a burden of responsibility on their shoulders:

*“[I said to [practitioner]] I’d rather you speak to me first before ya top yourself or something.” (Service User 2, Interview)*

#### Theme 3: Emotional impact of the disclosure.

**Emotional impact on the practitioner:** There was a striking mix of responses from practitioners in the survey about the perceived impact that the disclosure had on their wellbeing, with the most frequent response being ‘no impact’ (n = 34, 41%), followed by positive (n = 23, 27.7%) then mixed (n = 18, 21.7%) (S3 Table). Service users also had mixed views of the perceived emotional impact of the practitioner’s disclosure on the practitioner’s wellbeing, with more selecting ‘unsure’ (n = 20, 29.4%), followed closely by positive (n = 17, 25.0%) and ‘no impact’ (n = 16, 23.5%).

A mixed emotional impact on the practitioner was similarly captured within the interview narratives. One service user spoke of the disclosure seeming *“cathartic”* for the practitioner. Another service user reflected on a practitioner’s emotional resilience:

*“We think like nothing can get to them, but these people are human as well and they feel the emotions we do.” (Service User 5, Interview)*

Other service users felt their practitioner had not sought closure from their personal difficulties and disclosed as a result of emotional distress:

*“if you’ve overcome something you wouldn’t bring it up as much.” (Service User 1, Interview)*

A sense of apprehension following their disclosure was prevalent within some professional narratives, however there was a clear indication that overall disclosure had minimal impact on the practitioner’s wellbeing.

**Emotional impact on the service user:** In the survey, the vast majority of practitioners (n = 76, 91.6%) disagreed that their disclosure meant the service user was worried about telling them things in case they became upset, with only 4 (4.8%) agreeing with the statement. The disclosure had a mixed emotional impact on service users, with 38.2% (n = 26) agreeing that they did not want to know about their practitioner’s personal MH difficulties and 42.6% (n = 29) disagreeing indicating that they did want to know about their practitioner’s experiences (S4 Table). A majority of service user survey respondents (n = 40, 58.8%) agreed that they felt inspired by their practitioner’s recovery from MH difficulties, with 18 (26.5%) disagreeing.

Service user interviewees described the disclosure as a highly emotive experience, exemplified by the use of powerful descriptive language, such as *“healing experience”* and *“an amazing role model”.* One individual spoke with particular passion:

*“I’m getting really emotional talking about it to be honest [laughs] … having somebody there to inspire you and somebody who wants you to get well… it kinda accelerated my return to well-being.” (Service User 3, Interview)*

However, for others, the disclosure led them to judge their experiences against that of the professional, contributing to feelings of worthlessness and inferiority:

*“I’ve tried for… twenty, thirty years now to achieve things and I can’t get anywhere.” (Service User 2, Interview)*

#### Theme 4: Diagnosis and stigma.

The most frequently disclosed diagnoses reported by practitioners in the survey were anxiety (n = 52, 62.7%) and depression (n = 48, 57.8%), and these were also reported by service users to be the diagnoses most disclosed to them (n = 32, 47.1% and n = 31, 45.6% respectively). By comparison the next most frequently disclosed diagnosis was suicidal thoughts (n = 11, 13.3%) reported by practitioners and post-traumatic stress disorder (n = 11, 16.2%) reported as disclosed to them by service users (S5 Table).

Two-thirds (67.5%) of practitioners (n = 56) reported that the service user’s diagnosis influenced their decision to disclose, with only 24.1% (n = 20) saying they would have disclosed if the service user had a different diagnosis (S6 Table). In contrast, the majority of service users (n = 42, 61.8%) reported that they would feel the same way about the disclosure if the practitioner had disclosed any other diagnosis or set of symptoms, hence the commonality of diagnosis appeared less important to service users.

Analysis of the interview data highlighted a positive correlation between commonality of diagnosis and a more positive experience, particularly for practitioners. However, practitioners and service users opposed the idea that diagnosis was relevant, noting instead that the focus should be on presenting difficulties.

Stigma was also acknowledged across all narratives. Practitioners reported being unlikely to disclose their diagnosis if it were stigmatised by colleagues or service users, thus potentially making some diagnoses taboo. Some service users also inferred the stigma associated with certain conditions, describing diagnoses as a form of categorization and exclusion.

#### Theme 5: Professional roles, boundaries and the balance of power.

In the survey, the majority of service users (n = 43, 63.2%) stated they would feel the same if the disclosure was from a practitioner of a different professional background, with 25 (36.8%) strongly agreeing with this statement (S7 Table), indicating that things other than professional background (perhaps diagnosis, or rapport) were more important.

The only challenge to this narrative was highlighted in the interviews where there was a view by some that disclosure was not compatible with psychiatry. For example, psychiatrists were described by a practitioner as “*big, tough MDT professionals [of] … the upmost importance*” (Practitioner 1, Interview) and noted by a service user to be “*not allowed to kind of reach you on a human level*” (Service User 5, Interview).

The vast majority of practitioners (n = 70, 84.3%) and over half of service users (n = 36, 52.9%) disagreed that the disclosure had a negative effect on professional boundaries (S7 Table). However, whilst only one practitioner (1.2%) thought potential boundary crossing was problematic, this rose to 25 (36.8%) among service users, suggesting that service users valued professional boundaries more than practitioners, though still at a relatively low level.

Another consistent theme in the interviews was that of self-disclosure balancing the power dynamic between practitioners and service users and facilitating human interactions as expressed by a service user:

*“I did feel umm I’m not talking to a professional anymore, I’m talking to a human now, a real person now.” (Service User 3, Interview)*

One practitioner narrative criticised the unattainable perception of invincibility amongst practitioners and hinted at how disclosure helped to show service users that practitioners experienced difficulties too:

*“There may have been an assumption that because you’re a mental health social worker that our life is perfect…” (Practitioner 3, Interview)*

However, the difficulty of fully removing the power differentials was also alluded to:

*“You’ll never be equal, it’s impossible erm… because we’re coming from different angles.” (Practitioner 3, Interview)*

It was interesting to note this perception that ‘coming from different angles’ meant that individuals could not be equals.

#### Theme 6: Organisational context.

In the survey, more than half of all practitioners indicated that the culture of a team could impact on their willingness to self-disclose. Thirteen (15.7%) practitioners reported being very worried that their disclosure could be perceived negatively by their colleagues and manager, with 31 (37.3%) being a little worried. Those who had been in their role for longer appeared to be less concerned, perhaps due to greater confidence in their role or perceived value in the team.

In the interviews, inpatient, forensic and emergency settings were described as adding *“a whole new layer” (practitioner 5)* to disclosure in that they are “*controlled environments”* (service user 5) with limited links to the outside world and a focus on privacy and boundaries. Unlike in community settings where a practitioner disclosure was unlikely to be shared and discussed between service users, inpatient and forensic settings could see disclosures being shared between patients on wards and the practitioner losing any control of who heard the disclosure, including colleagues and managers.

A progressive movement from ‘*us and them’ to* celebrating lived experience was identified by service users who had accessed services for a number of years. This was also reflected by a practitioner who suggested establishing a *“new norm”* surrounding disclosure in services:

*“I am so big on having open conversations in the office and the more we talk about something, the more it becomes a part of our culture.” (Practitioner 2)*

However, practitioners highlighted the need for guidance, supervision and mental health support for practitioners around disclosure, particularly for newly qualified staff.

## Discussion

This research was undertaken to offer greater insight into service users’ and mental health practitioners’ views and experiences around practitioners’ self-disclosure of mental health difficulties. While quantitative survey data provided the bigger picture, analysis of open-ended survey questions and qualitative interviews helped to explore views and experiences in more depth and also explain and interpret the figures. The study found that practitioners’ self-disclosure can provide a valuable contribution to service users’ care. Self-disclosure offers benefits for both practitioners and service users, such as promoting recovery, combatting stigma and balancing the power differentials. However, there is a notable lack of reflective supervision, clear guidance and pastoral support for practitioners around disclosure. Furthermore, practitioner self-disclosure can be misunderstood by service users. The findings suggest that practitioners are more likely to disclose the longer they have been practising, suggesting that team culture, confidence and professional capability are influential.

The findings indicate that mental health practitioners’ rationale for disclosure of their own mental health problems to service users is to generate partnership and empathy with service users and thereby better support service users. This is consistent with other studies [12,13] and further contributes to the evidence base as there is a lack of clarity around practitioners’ rationales for disclosure. Previous studies proposed that disclosure violates boundaries which can result in service users having less confidence in the practitioner’s capabilities [5,17,18]. However, the current findings demonstrated that, for most participants, self-disclosure was not perceived to cross boundaries and determined that practitioner confidence and capability correlated with more disclosures. Context, timing and presentation were vital factors for practitioners to consider prior to disclosing since service users who were in a difficult place in terms of their recovery were less able to listen to their practitioner’s personal issues.

This research contributes to the evidence base, suggesting that first-hand experience is valued by service users over textbook knowledge or a professional *“script*” as it demonstrates vulnerability and sensitivity [8]. However, the research also identified a number of disadvantages in which disclosure can contribute to a shift in focus from service user to practitioner, reinforcing the perspectives of traditional psychoanalysts [7]. The research suggests that disclosure can cause service users to make an unhelpful comparison between recovery journeys, an area largely unexplored.

Furthermore, the research indicates that practitioners’ wellbeing was largely unaffected by their disclosure while, for most service users, it was an emotive experience which instilled hope and recovery. The majority of service users broadly supported practitioners’ sharing lived experience [14] and found that self-disclosure helped to promote empowerment and address the power imbalance between practitioners and service users [10,11]. Both populations appreciated the value in breaking down the perception of invincibility among practitioners. The research established that a commonality of diagnosis correlated with a more positive experience. However, stigma continued to permeate organisational and social structures, with some practitioners fearful of disclosing due to anticipated stigma and negative repercussions within the workplace [38].

Previous research indicated that psychologists, occupational therapists and counsellors were more accepting of self-disclosure, whereas nurses, doctors, and social workers viewed self-disclosure as risky and unhelpful [14]. This research counters previous findings as self-disclosure was utilised across all professions, namely nursing, support work and psychology. Service users agreed that the professional background of the practitioner was immaterial, except for psychiatry. Notably, practitioners and service users identified psychiatry as incompatible with self-disclosure due to psychiatrists’ medical professional status, supporting previous research findings [16]. However, the current research found that a number of psychiatrists self-disclose frequently within their practice which suggests a discrepancy between the way psychiatrists perceive and are perceived to perceive self-disclosure.

Overall, mental health practitioners’ disclosure of their own mental health difficulties to the service users they work with was reported as broadly beneficial by both practitioners and service users. However, this is a personal act by the practitioner and not all service users welcomed the disclosure thus care and caution need to be taken by practitioners in determining if, when and how to disclose. Guidance and support for practitioners would thus be helpful.

### Limitations

Recruitment via online channels and links to specific groups/networks could have excluded some sections of the target populations. There was a skew toward community mental health professionals taking part in the survey thus caution must be taken when interpreting the findings. As discussed, data on race and ethnicity, on gender and sexual identity was not collected and there was a lack of diversity in recruitment. This is both a limitation of the current study and a subject for future study - how racism, heterosexism, cissexism, transphobia, for example, shape self-disclosures and how people make sense of them. The disruption caused by COVID-19 placed additional strains on frontline practitioners and members of the public, likely contributing to a smaller sample size which limits the generalisability of the results. The emotive nature of the topic may also have prevented some eligible people from taking part. The study findings and implications for policy and practice thus need to be read with caution.

### Implications for policy and practice

The research indicates that stigma is widespread within mental health settings. Service users have clearly identified that practitioners with lived experience of mental health difficulties provide a valuable contribution to the care they receive. A key suggestion is thus bringing to the forefront of the mental health workforce recognition of the skillset and knowledge that practitioners with lived experience possess in order to embed voluntary self-disclosure within teams and organisations. The research suggests that practitioners must feel secure and supported within the workplace when choosing to self-disclose. A notable lack of guidance, supervision and pastoral support in facilitating this complex and controversial process results in self-disclosure taking place often unobserved and with a lack of confidence, particularly amongst newly qualified staff or within teams with a lack of progression. Offering all mental health practitioners training, guidance and reflective supervision around self-disclosure is thus likely to increase professional confidence and reduce the sense of invisibility around disclosure. Involving experts by experience and peer support practitioners to facilitate open discussions around successful and unsuccessful experiences of self-disclosure could be useful.

The findings also suggest that whilst practitioners have a clear rationale for their disclosure - to promote recovery, instil hope and validate service users’ experiences - the aim of the disclosure is sometimes misunderstood or unclear from the perspective of the service user. A further suggestion would thus be for a discussion on self-disclosure to take place either before or shortly after disclosure to provide clarity.

To conclude, training, guidance, reflective supervision and utilising the knowledge of those with lived experience of mental health problems could increase practitioner confidence in decision-making around disclosure of their own mental health problems to service users to further support service users on their roads to recovery, support practitioners’ own mental health and to further help reduce stigma.

## Supporting information

S1 Table Practitioners’ and service users’ views on the short-term and long-term benefits of disclosure. (DOCX)

S2 Table Practitioners’ and service users’ views on whether service users felt judged by practitioners after they disclosed their own MH difficulties. (DOCX)

S3 Table Practitioners’ and service users’ views on the impact of the practitioner’s disclosure on the practitioner’s wellbeing. (DOCX)

S4 Table Practitioners’ and service users’ views on the impact of the practitioner’s disclosure on the service user’s wellbeing. (DOCX)

S5 Table Mental health diagnoses/symptoms disclosed to service users by practitioners. (DOCX)

S6 Table Practitioner perspectives on the influence of the service user’s diagnosis on the disclosure. (DOCX)

S7 Table Practitioner and service user views on professional boundaries. (DOCX)
