# Supplementary material for: To disclose or not to disclose? Mental health service users’ and practitioners’ views of practitioners’ own self-disclosure of mental health difficulties: A mixed-methods study
Source: PLOS Ment Health. 2025 Apr 8;2(4):e0000062. doi: 10.1371/journal.pmen.0000062 (PMC12798165; doi:10.1371/journal.pmen.0000062)
Supplement: S1 Table — (DOCX) [file pmen.0000062.s001.docx]

S1 Table: Practitioners’ and service users’ views on the short-term and long-term benefits of disclosure

|  | Yes, a lot  n(%) | Yes, a little  n(%) | No, not at all  n(%) | Unsure  n(%) |
| --- | --- | --- | --- | --- |
| **Practitioners (n=83): Do you think your disclosure benefitted the service user:** | | | | |
| In the short term | 42(50.6) | 35(42.2) | 1(1.2) | 5(6.0) |
| In the longer term | 24(28.9) | 36(43.4) | 1(1.2) | 22(26.5) |
| **Service users (n=68): Do you think the practitioner’s disclosure was a benefit to you:** | | | | |
| In the short term | 21(30.9) | 23(33.8) | 16(23.5) | 8(11.8) |
| In the longer term | 19(27.9) | 20(29.4) | 21(30.9) | 8(11.8) |
