# Supplementary material for: To disclose or not to disclose? Mental health service users’ and practitioners’ views of practitioners’ own self-disclosure of mental health difficulties: A mixed-methods study
Source: PLOS Ment Health. 2025 Apr 8;2(4):e0000062. doi: 10.1371/journal.pmen.0000062 (PMC12798165; doi:10.1371/journal.pmen.0000062)
Supplement: S2 Table — (DOCX) [file pmen.0000062.s002.docx]

S2 Table: Practitioners’ and service users’ views on whether service users felt judged by practitioners after they disclosed their own MH difficulties

|  | Strongly agree  n(%) | Agree  n(%) | Somewhat agree  n(%) | Neither agree nor disagree  n(%) | Disagree  n(%) | Somewhat disagree  n(%) | Strongly disagree  n(%) |
| --- | --- | --- | --- | --- | --- | --- | --- |
| **Service user views (n=68):** | | | | | | | |
| I felt that the practitioner thought I should be doing better in my own recovery journey | 8(11.8) | 6(8.8) | 12(17.6) | 10(14.7) | 12(17.6) | 4(5.9) | 16(23.5) |
| I think the practitioner judged my recovery journey by comparing it to their own | 12(17.6) | 7(10.3) | 9(13.2) | 14(20.6) | 8(11.8) | 4(5.9) | 14(20.6) |
| **Practitioner views (n=83):** | | | | | | | |
| I think the service user was worried that I felt they should be doing better in terms of their own recovery journey | 0 | 1(1.2) | 6(7.2) | 8(9.6) | 31(37.7) | 8(9.6) | 29(34.9) |
| I judged the service user’s recovery journey compared to my own mental health recovery | 2(2.4) | 2(2.4) | 4(4.8) | 7(8.4) | 24(28.9) | 6(7.2) | 38(45.9) |
