# Supplementary material for: To disclose or not to disclose? Mental health service users’ and practitioners’ views of practitioners’ own self-disclosure of mental health difficulties: A mixed-methods study
Source: PLOS Ment Health. 2025 Apr 8;2(4):e0000062. doi: 10.1371/journal.pmen.0000062 (PMC12798165; doi:10.1371/journal.pmen.0000062)
Supplement: S3 Table — (DOCX) [file pmen.0000062.s003.docx]

S3 Table: Practitioners’ and service users’ views on the impact of the practitioner’s disclosure on the practitioner’s wellbeing

|  | Positive  n(%) | Negative  n(%) | No impact  n(%) | Unsure  n(%) | Mixed  n(%) | Other  n(%) |
| --- | --- | --- | --- | --- | --- | --- |
| **Practitioner views (n=83):** | | | | | | |
| What impact did your disclosure have on your own wellbeing? | 23(27.7) | 1(1.2) | 34(41.0) | 3(3.6) | 18(21.7) | 4(4.8) |
| **Service user views (n=68):** | | | | | | |
| What impact do you think the practitioner’s disclosure had on their own wellbeing? | 17(25.0) | 3(4.4) | 16(23.5) | 20(29.4) | 12(17.6) | 0 |
