# Supplementary material for: To disclose or not to disclose? Mental health service users’ and practitioners’ views of practitioners’ own self-disclosure of mental health difficulties: A mixed-methods study
Source: PLOS Ment Health. 2025 Apr 8;2(4):e0000062. doi: 10.1371/journal.pmen.0000062 (PMC12798165; doi:10.1371/journal.pmen.0000062)
Supplement: S4 Table — (DOCX) [file pmen.0000062.s004.docx]

S4 Table: Practitioners’ and service users’ views on the impact of the practitioner’s disclosure on the service user’s wellbeing

|  | Strongly agree  n(%) | Agree  n(%) | Somewhat agree  n(%) | Neither agree nor disagree  n(%) | Disagree  n(%) | Somewhat disagree  n(%) | Strongly disagree  n(%) |
| --- | --- | --- | --- | --- | --- | --- | --- |
| **Practitioner views (n=83):** | | | | | | |  |
| I don’t think the service user wanted to know about my personal MH difficulties | 2(2.4) | 2(2.4) | 1(1.2) | 25(30.1) | 22(26.5) | 21(25.3) | 10(12.0) |
| Following my disclosure, I think the service user was worried about telling me some things in case I became upset | 1(1.2) | 0 | 3(3.6) | 3(3.6) | 29(34.9) | 6(7.2) | 41(49.4) |
| **Service user views (n=68):** | | | | | | |  |
| I didn’t want to know about my practitioner’s personal MH difficulties | 8(11.8) | 11(16.2) | 7(10.3) | 13(19.1) | 11(16.2) | 6(8.8) | 12(17.6) |
| Following their disclosure, I worried about telling the practitioner some things in case they became upset | 14(20.6) | 6(8.8) | 4(5.9) | 5(7.4) | 10(14.7) | 5(7.4) | 24(35.3) |
| I felt inspired by the practitioner’s recovery from MH difficulties | 21(30.9) | 8(11.8) | 11(16.2) | 10(14.7) | 8(11.8) | 2(2.9) | 8(11.8) |
