# Supplementary material for: To disclose or not to disclose? Mental health service users’ and practitioners’ views of practitioners’ own self-disclosure of mental health difficulties: A mixed-methods study
Source: PLOS Ment Health. 2025 Apr 8;2(4):e0000062. doi: 10.1371/journal.pmen.0000062 (PMC12798165; doi:10.1371/journal.pmen.0000062)
Supplement: S5 Table — (DOCX) [file pmen.0000062.s005.docx]

S5 Table: Mental health diagnoses/symptoms disclosed to service users by practitioners

| **Diagnosis/symptoms** | **What practitioners stated they disclosed (n=83)**  **n(%)** | **What service users stated was disclosed to them (n=68)**  **n(%)** |
| --- | --- | --- |
| Anxiety | 52(62.7) | 32(47.1) |
| Bipolar disorder | 2(2.4) | 3(4.4) |
| Depression | 48(57.8) | 31(45.6) |
| Eating disorder | 5(6.0) | 7(10.3) |
| Obsessive compulsive disorder | 2(2.4) | 3(4.4) |
| Personality disorder | 3(3.6) | 8(11.8) |
| Post-traumatic stress disorder | 5(6.0) | 11(16.2) |
| Psychosis | 0 | 1(1.5) |
| Schizophrenia | 0 | 1(1.5) |
| Self-harm | 8(9.6) | 6(8.8) |
| Suicidal thoughts | 11(13.3) | 10(14.7) |
| Other | 9(10.8) | 11(16.2) |
