# Supplementary material for: To disclose or not to disclose? Mental health service users’ and practitioners’ views of practitioners’ own self-disclosure of mental health difficulties: A mixed-methods study
Source: PLOS Ment Health. 2025 Apr 8;2(4):e0000062. doi: 10.1371/journal.pmen.0000062 (PMC12798165; doi:10.1371/journal.pmen.0000062)
Supplement: S6 Table — (DOCX) [file pmen.0000062.s006.docx]

S6 Table: Practitioner perspectives on the influence of the service user’s diagnosis on the disclosure

|  | Yes, definitely  n(%) | Yes, probably  n(%) | Might or might not  n(%) | Probably not  n(%) | Definitely not  n(%) |
| --- | --- | --- | --- | --- | --- |
| **Practitioner views (n=83):** | | | | | |
| Do you feel that the service user’s diagnosis had any influence on your decision to disclose? | 21(25.3) | 35(42.2) | 7(8.4) | 12(14.5) | 8(9.6) |
| Would you have made the disclosure if the service user had a different diagnosis/ symptoms? | 6(7.2) | 14(16.9) | 28(33.7) | 26(31.3) | 9(10.8) |
| **Service user views (n=68):** | | | | | |
| I would feel the same if the practitioner had disclosed any other MH diagnosis/symptoms | 18(26.5) | 24(35.3) | 14(20.6) | 9(13.2) | 3(4.4) |
