# Supplementary material for: To disclose or not to disclose? Mental health service users’ and practitioners’ views of practitioners’ own self-disclosure of mental health difficulties: A mixed-methods study
Source: PLOS Ment Health. 2025 Apr 8;2(4):e0000062. doi: 10.1371/journal.pmen.0000062 (PMC12798165; doi:10.1371/journal.pmen.0000062)
Supplement: S7 Table — (DOCX) [file pmen.0000062.s007.docx]

S7 Table: Practitioner and service user views on professional boundaries

|  | Strongly agree  n(%) | Agree  n(%) | Somewhat agree  n(%) | Neither agree nor disagree  n(%) | Disagree  n(%) | Somewhat disagree  n(%) | Strongly disagree  n(%) |
| --- | --- | --- | --- | --- | --- | --- | --- |
| **Service user views (n=68):** | | | | | | | |
| I would feel the same if the disclosure was from a practitioner from a different profession | 25(36.8) | 14(20.6) | 4(5.9) | 8(11.8) | 10(14.7) | 3(4.4) | 4(5.9) |
| I think that the practitioner crossed a line that they should not have done | 11(16.2) | 8(11.8) | 6(8.8) | 7(10.3) | 9(13.2) | 6(8.8) | 21(30.9) |
| **Practitioner views (n=83):** | | | | | | | |
| My disclosure had a negative impact on my professional boundaries with the service user | 0 | 0 | 1(1.2) | 12(14.5) | 27(32.5) | 9(10.8) | 34(41.0) |
